# Emerging trends and hotspots evolution in cardiotoxicity: A bibliometric and knowledge-Map analysis From 2010 to 2022

**DOI:** 10.3389/fcvm.2023.1089916

**Published:** 2023-03-07

**Authors:** Di Xiao, Jingen Li, Yong Liu, Tangshun Wang, Chaofeng Niu, Rui Zhuang, Birong Liu, Liyong Ma, Meng Li, Lijing Zhang

**Affiliations:** ^1^ Department of Cardiology, Dongzhimen Hospital, Beijing University of Chinese Medicine, Beijing, China; ^2^ Department of Cardiology, Dongfang Hospital, Beijing University of Chinese Medicine, Beijing, China; ^3^ Department of General Surgery, Dongzhimen Hospital, Beijing University of Chinese Medicine, Beijing, China

**Keywords:** cardiotoxicity, bibliometric, knowledge-map, citespace, VOSviewer

## Abstract

**Background:**

There is growing emphasis on the cardiotoxicity research over the past 12 years. To look for the hotspots evolution and to explore the emerging trends in the field of cardiotoxicity, publications related to cardiotoxicity were acquired from the Web of Science Core Collection on August 2, 2022.

**Methods:**

We used the CiteSpace 5.8 R3 and VOSviewer 1.6.18 to perform bibliometric and knowledge-map analysis.

**Results:**

A total of 8,074 studies by 39,071 authors from 6,530 institutions in 124 countries or regions were published in different academic journals. The most productive country was absolutely the United States, and the University of Texas MD Anderson Cancer Center was the institution with the largest output. Zhang, Yun published the most articles, and the author who had the most frequent co-citations was Moslehi, Javid. New England Journal of Medicine was the most frequently cited journals in this field. Mechanisms of cardiotoxicity have received the most attention and was the main research directions in the field. The disease of cardiotoxicity together with the related risk factors are potential research hotspots. Immune checkpoint inhibitor and myocarditis are two recently discussed and rapidly expanding research topic in the areas of cardiotoxicity.

**Conclusions:**

This bibliometric analysis provided a thorough analysis of the cardiotoxicity, which would provide crucial sources of information and concepts for academics studying this area. As a rapidly expanding field in cardiology, the related field of cardiotoxicity will continue to be a focus of research.

## Introduction

Cardiotoxicity, referring to the direct adverse effect of cancer treatment on function or structure of the heart, is the most common side effect of cancer therapy. Cardiotoxicity from cancer therapy may occur either during cancer treatment or many years after, which can cause cardiac dysfunction and heart failure (HF), coronary heart disease, arrhythmias, cardiomyopathy, myocarditis and pericarditis (1). It is causing increasing concerns that cardiotoxicity may lead to premature death from cardiovascular disease among cancer survivors (2). Nearly 14.5 million children and adults are long-term survivors of cancer (more than 5 years) according to a 2014 American Cancer Society study, and this number is expected to grow to 19 million by 2024 (3). As this population steadily increases, long-term secondary side effects associated with cancer therapy become more significant. Actually, the increasing incidence of cardiotoxicity has promoted the development of a multidisciplinary field called cardio-oncology (4). In addition, new cancer drugs are constantly being developed, but their uncertain cardiotoxicity makes long-term surveillance and care challenging. Due to its great potential, cardiotoxicity has attracted the attention of scholars to gain a deeper understanding of the process of cardiotoxicity and the challenges and opportunities surrounding it. Over the past 12 years, there had been a growing body of clinical and basic research on cardiotoxicity (5, 6). Scholars and clinicians find it increasingly challenging, even within their specialties, to keep up with the latest advances with the growing number of publications. While systematic reviews and meta-analyses can be of great help in understanding some specific research questions in the area of cardiotoxicity (7–10), it can't summarize current research frontiers, or display high-influencing labs or institutions in the area of cardiotoxicity.

Bibliometrics is the qualitative and quantitative analysis of academic publication and thus can evaluate emerging trends and describe relationships between published works (11–14). Bibliometric methods are expected to complement qualitative structured literature reviews and meta-analyses by providing new insights into the reviews and evaluations of scientific literature on a more intuitive level. Based on its strengths, it is of great value in assessing research trends and formulating guidelines (15). However, no bibliometric analysis has provided a thorough analysis of cardiotoxicity.

Thus, we used bibliometrics analysis to objectively assess recent developments in the field of cardiotoxicity research over the past 12 years from the following three different perspectives: (i) We planned to identify and quantify general data on cardiotoxicity research, including information on individual contributions and collaborations, by analyzing annual publications, countries, institutions, journals, co-cited journals, authors, and co-cited authors. (ii) We designed to assess the knowledge base of cardiotoxicity by examining the most co-cited articles through a co-cited reference analysis. (iii) Finally, but most importantly, by analyzing co-cited reference bursts and keywords, we identified emerging topics in cardiotoxicity and discover the knowledge structure and hotspot evolution. Overall, our objective study aims to provide the first comprehensive assessment and analysis of cardiotoxicity from 2010 to 2022 based on bibliometric analysis. It will provide fresh insights for researchers on the current status, the development trends, and research hotspots in the cardiotoxicity field.

## Materials and methods

### Data collection and search strategy

The Web of Science Core Collection (WoSCC) database is widely used in bibliometric analysis (16–18). Aside from its comprehensive bibliometric software capabilities, we also chose it for its reputation as the most influential database when it comes to bibliometric software (19). Web of Science (WoS, Clarivate Analytics, Philadelphia, PA, United States), which contains more than 12,000 international academic journals, is one of the most comprehensive and authoritative database platforms to obtain global academic information. Apart from the general literature search, it also possesses an important function of citation index searching, which is helpful for assessing the academic performance of literature in a specific field (20, 21).

We retrieved the data from WoSCC database on August 2, 2022. The search strategy was (TS = [cardiotoxicity or cardiovascular toxicities or cardiac toxicity or Myocardial toxicity]), and the publication year was limited to 2010- 2022. There was no language limit, and the publication type was limited to only Article or Review. We downloaded all retrieved records together with their cited References in the format of plain text. The files were then transformed to text file with the name of “download *.txt” which could be recognized by Citespace software.

### Data analysis and visualization

For bibliometric analysis and visualization, we used CiteSpace (version 5.8.R3 [Chaomei Chen, 2006]), VOSviewer (version 1.6.18 [Nees Jan van Eck and Ludo Waltman, 2010]) to analyze the data and create a visual representation of scientific knowledge. The annual publications were analyzed and managed using Microsoft Office Excel 2019. We derived the annual growth trend of publication outputs by analyzing the data and producing the figures using Microsoft Office Excel 2019.

As a commonly used visualization software in bibliometrics, CiteSpace is capable of detecting collaborations, hot spots, internal structures, possible trends, and evolutionary processes in scientific fields [22]. Therefore, CiteSpace was used to generate visual map of distribution of countries/regions and institutions, author, the dual-map of journals, reference timelines, and keyword citation bursts. We choose the following settings: time span is set to 2010–2022 and years per slice is 1; Top *N* = 50 filters the top 50 authors, organizations, and keywords with the highest frequency in each time slice; the pruning is set to Pathfinder. With the CiteSpace visualization, nodes are sized according to the frequency of co-occurrences, and links show the relationship between co-occurrences. The colors of the nodes and lines change from purple to red as the year 2010 to 2022 progresses. Purple round nodes have a strong centrality (≥0.10), which serves as a role of bridge interconnecting various networks through it (22–24).

As another bibliometric tool, VOSviewer can create and visualize knowledge maps, showing clusters, timeline, or density colors (25, 26). We used it to detect citation of country/region and institution collaboration, as well as co-cited authors, productive journals, and keywords. Data were analyzed using VOSviewer based on the full counting method. Cluster maps represented co-occurrence frequencies based on node size, and nodes of the same color were in the same cluster. In addition, the thickness of links indicated co-occurrence relations that were defined by their strength, which depends on how often two researchers co-authored papers or how frequently two keywords appeared together in papers (26). The co-citation frequency was positively correlated with word size, circular size, and yellow opacity in the density map. The overlay map displays the average publication year in different colors. Additionally, we obtained the impact factor (IF) and Journal citation reports (JCR) divisions of journals on August 2, 2022 from Web of Science.

## Results

### The annual growth trend of publication outputs

**Figure 1:**
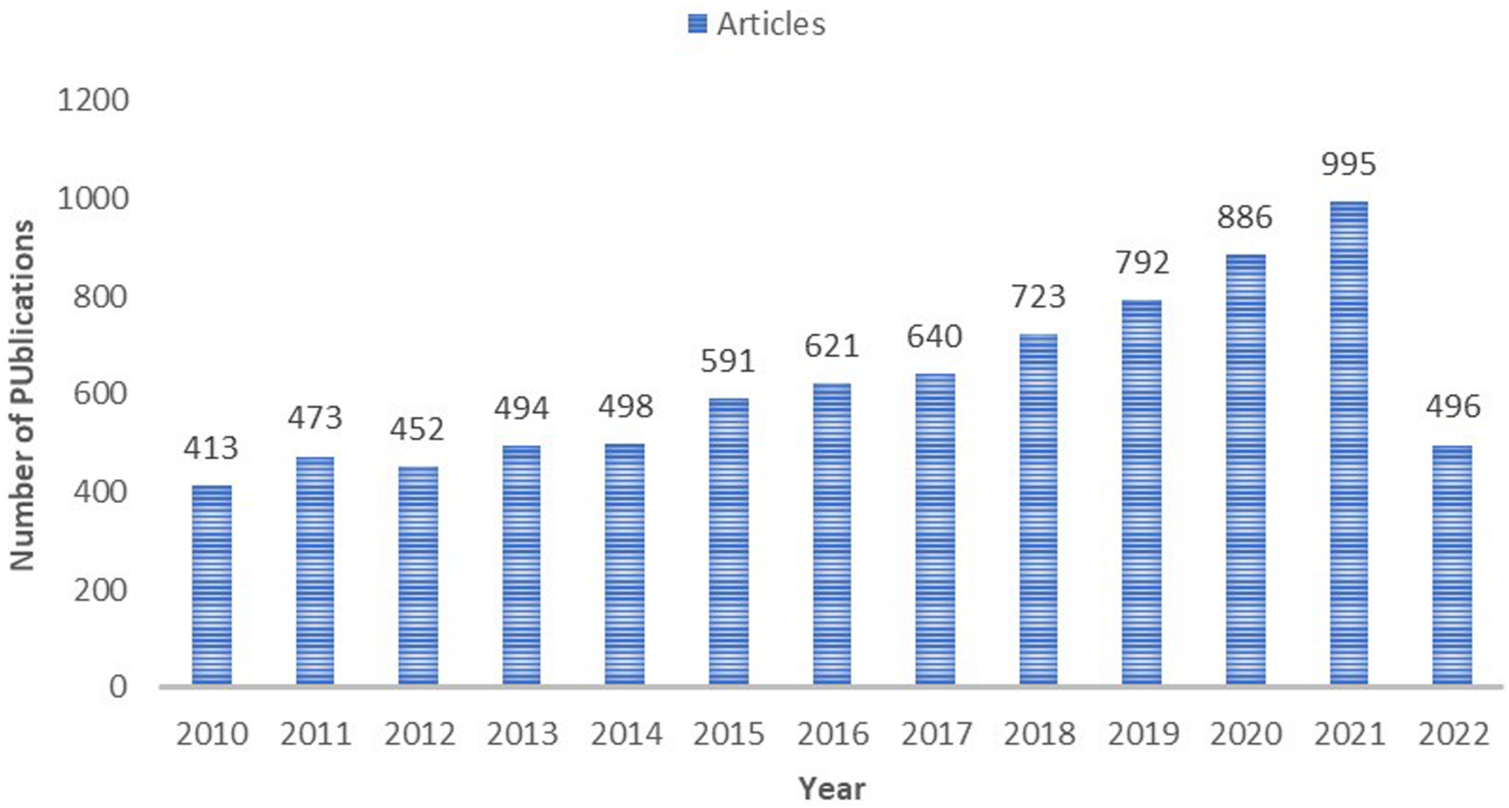
The annual growth trend of publication outputs.

Among the 8,077 papers we obtained from the WoSCC database, we eventually included 8,074, after removing duplicate papers. Overall, there was an increase in the number of publications each year. Considering Figure 1, the history of research can be divided into three stages: (i) 2010–2013: in the early stages, during which 400–500 publications were published annually. (i) 2014–2018: in the smooth growth stages, during which 500–800 publications were published annually. (iii) 2019–2022: Rapid development phase. The number of papers on cardiotoxicity exceeded 800 in 2020.

### Countries/regions

**Figure 2:**
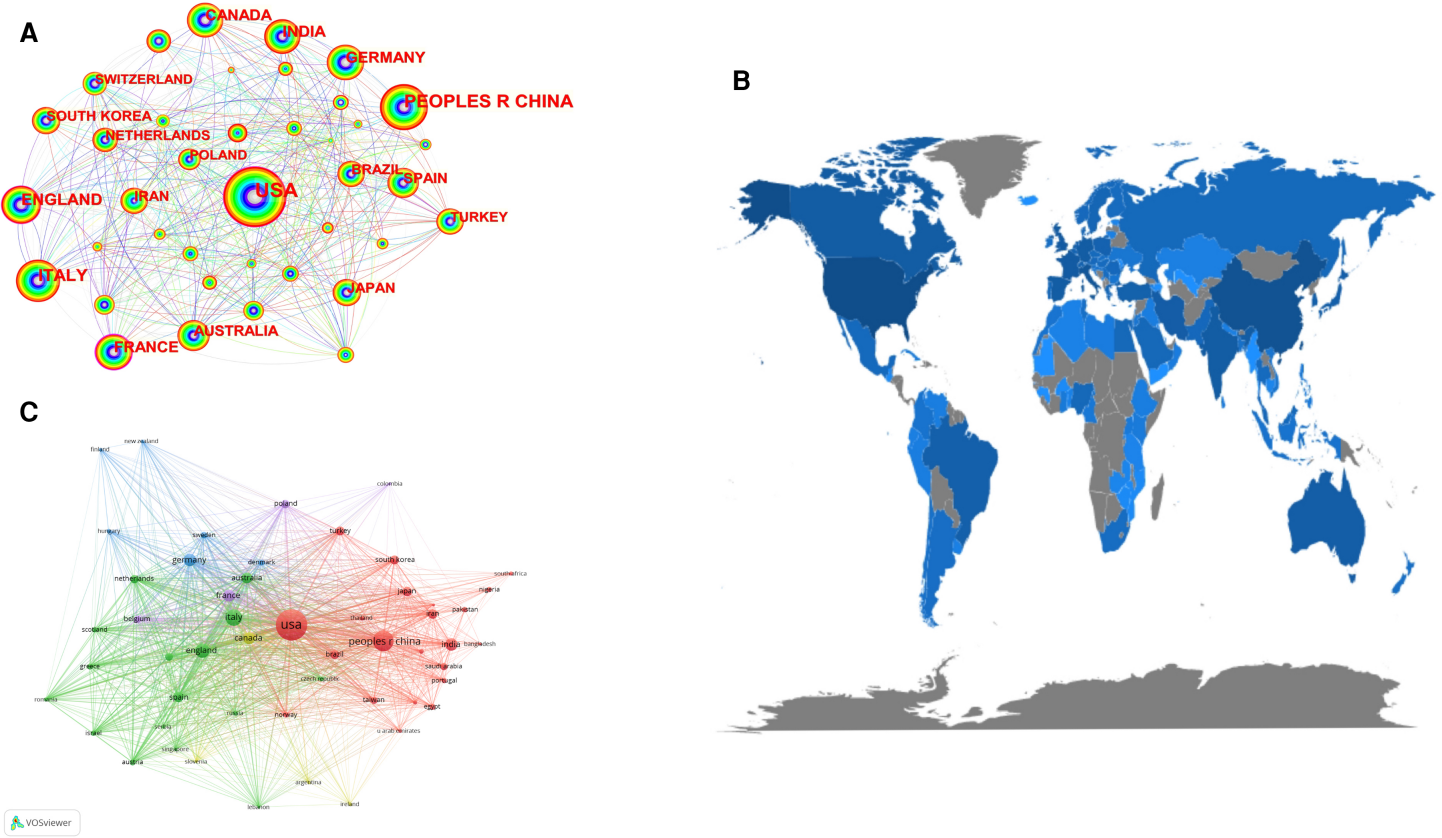
(**A**) the co-occurrence map of countries/regions in cardiotoxicity. In the network map, a node represents a country/region and the node size is proportionate to the quantity of publications produced by that country/region. (**B**) World map displaying the global distribution of cardiotoxicity research. Different countries were denoted with different colors based on the number of total citations. (**C**) Citation map of countries/regions on cardiotoxicity research generated by the VOS viewer. Each node represents a different country or region, and the node size is proportionate to the quantity of publications produced by that country. The distance between two nodes indicates the relatedness of their co-authorship link, and a smaller distance implies a stronger correlation of their relatedness. The thickness of the connecting line between nodes indicates link strength of a co-authorship relationship, which could be weighted by a quantitative indicator, that is, TLS.

A total of 8,074 total publications (TP) were published from 124 countries or regions between 2010 and 2022, with 69% of the total publications coming from the top 10 countries(Table 1). Table 1, Figure 2A shows that most of the articles were from the United States (2,178 articles, 27%), followed by China (1,121 articles, 13.9%), Italy (559, 6.7%), India (338, 4.2%), and England (305, 3.8%). Among all countries, total citations(TC = 81,970) were highest in the United States, indicating its leadership in cardiovascular toxicology. In Figure 2B, the total number of citations for the global article is shown.

**Table 1:** The top 10 countries/regions involved in cardiotoxicity.

Rank	Country	TP	Centrality	Percentage (*n* = 8074)	TC	TLS
1	United States	2178	0.14	0.27	81,970	10,594
2	CHINA	1121	0.01	0.139	21,972	2373
3	ITALY	559	0.05	0.069	23,187	4977
4	INDIA	338	0.09	0.042	7245	545
5	ENGLAND	305	0.16	0.038	22,994	3633
6	FRANCE	259	0.26	0.032	11,215	2047
7	CANADA	241	0.04	0.03	16,924	2408
8	GERMANY	232	0.09	0.029	13,770	2000
9	IRAN	200	0.01	0.025	5625	495
10	AUSTRALIA	178	0.06	0.022	2493	890

TP, Total publications; TC, Total number of citations of total publications; TLS, Total linkstrength.

In cardiovascular toxicology, the United States led the way, working with Italy and England most closely. On the citation network map (Figure 2C), we visualized the citation relationships between countries/regions. The VOSviewer parameter was set to have a minimum of 50 documents per country, and we received 60 threshold. Clearly, the United States had the strongest total link strength (TLS = 10,594), with close ties to Italy, England, and Canada.

### Contributions of institutions

**Figure 3:**
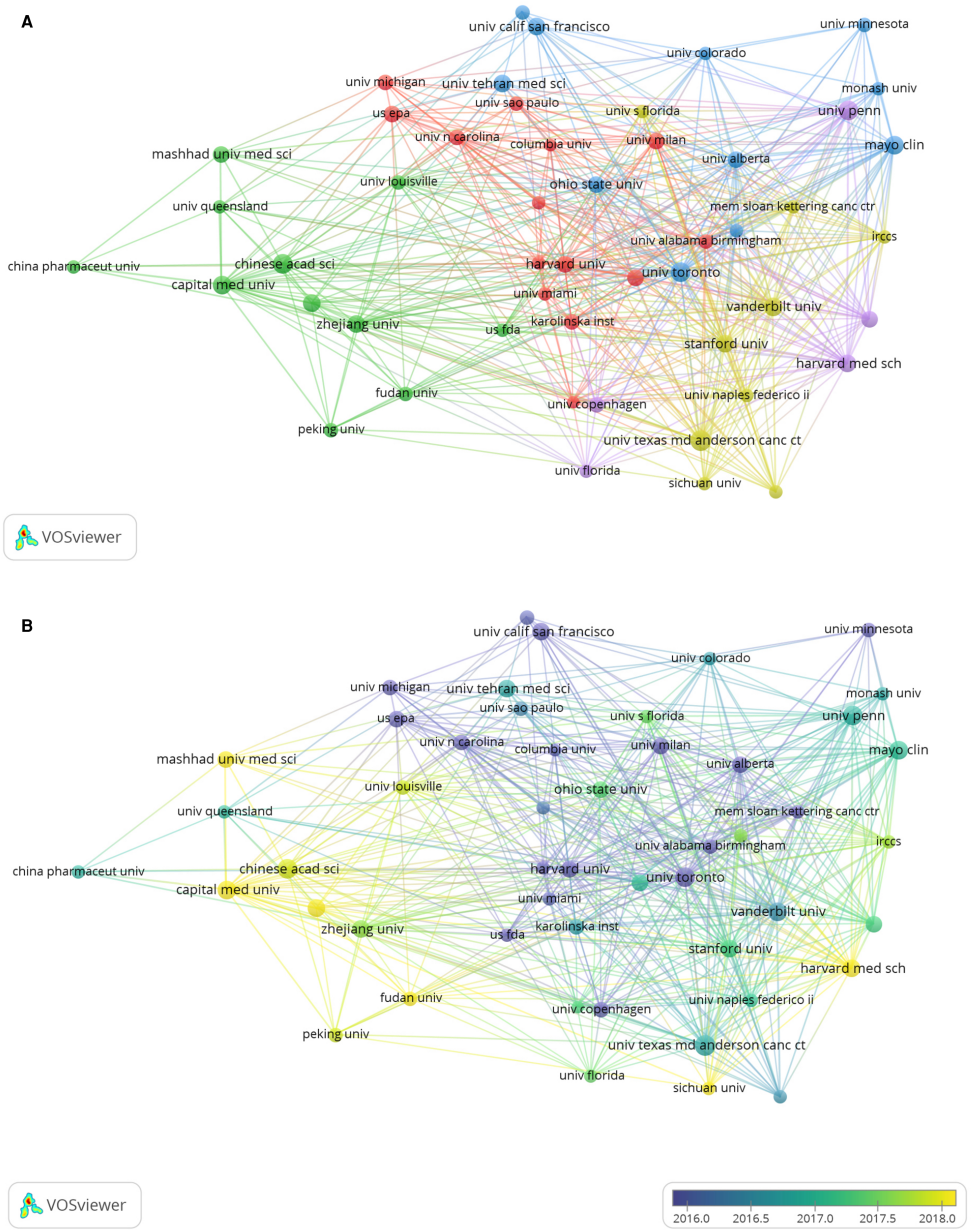
(**A**) network map of institution collaboration analysis based on VOSviewe. (**B**) Network map of institution collaboration analysis based on VOSviewer. Each node represents a different institution, and the node size is proportionate to the quantity of publications produced by that institution. The distance between two nodes indicates the relatedness of their collaboration link, and a smaller distance implies a stronger correlation of their relatedness. The thickness of the connecting line between nodes indicates link strength of a collaboration relationship, which could be weighted by a quantitative indicator, that is, TLS.

A total of 8,074 publications were published by 6,530 institutions. Table 2 shows the ten most productive institutions. Among them, The University of Texas MD Anderson Cancer Center (TP = 62) was the most prolific institution, followed by University of Toronto (TP = 56) and University of Pennsylvania (TP = 55). By using VOS viewer, we were able to visualize the network of institutions with more than 25 publications. The collaboration between institutions is shown in Figure 3A, containing 52 items grouped into five clusters of different colors. First placed was Vanderbilt University with 166 TLS, Harvard Medical School (TLS = 115) and Mayo Clinic (TLS = 106) ranked second and third, respectively. We then colored the institutions based on when they began to do research on cardiotoxicity, the bluer their colors, the earlier they started and the yellower the color, the later the institution appeared. Harvard Medical School and The University of Texas MD Anderson Cancer Center were pioneers in the field, as illustrated in Figure 3B.

**Table 2:** The top 10 institutions involved in cardiotoxicity.

Rank	Institutions	TP	TC	TLS
1	Vanderbilt University	49	2375	166
2	Harvard Medical School	45	1599	115
3	Mayo Clinic	51	1185	106
4	Capital medicine university	53	1433	101
5	University of Toronto	56	2582	94
6	The University of Texas MD Anderson Cancer Center	62	1421	93
7	Stanford University	44	1809	92
8	University of Pennsylvania	55	2635	82
9	Brigham and women's hospital	40	1018	70
10	University-of-Naples-Federico	29	903	60

TP, Total publications; TC, Total number of citations of total publications; TLS, Total linkstrength.

### Authors and co-cited authors

**Figure 4:**
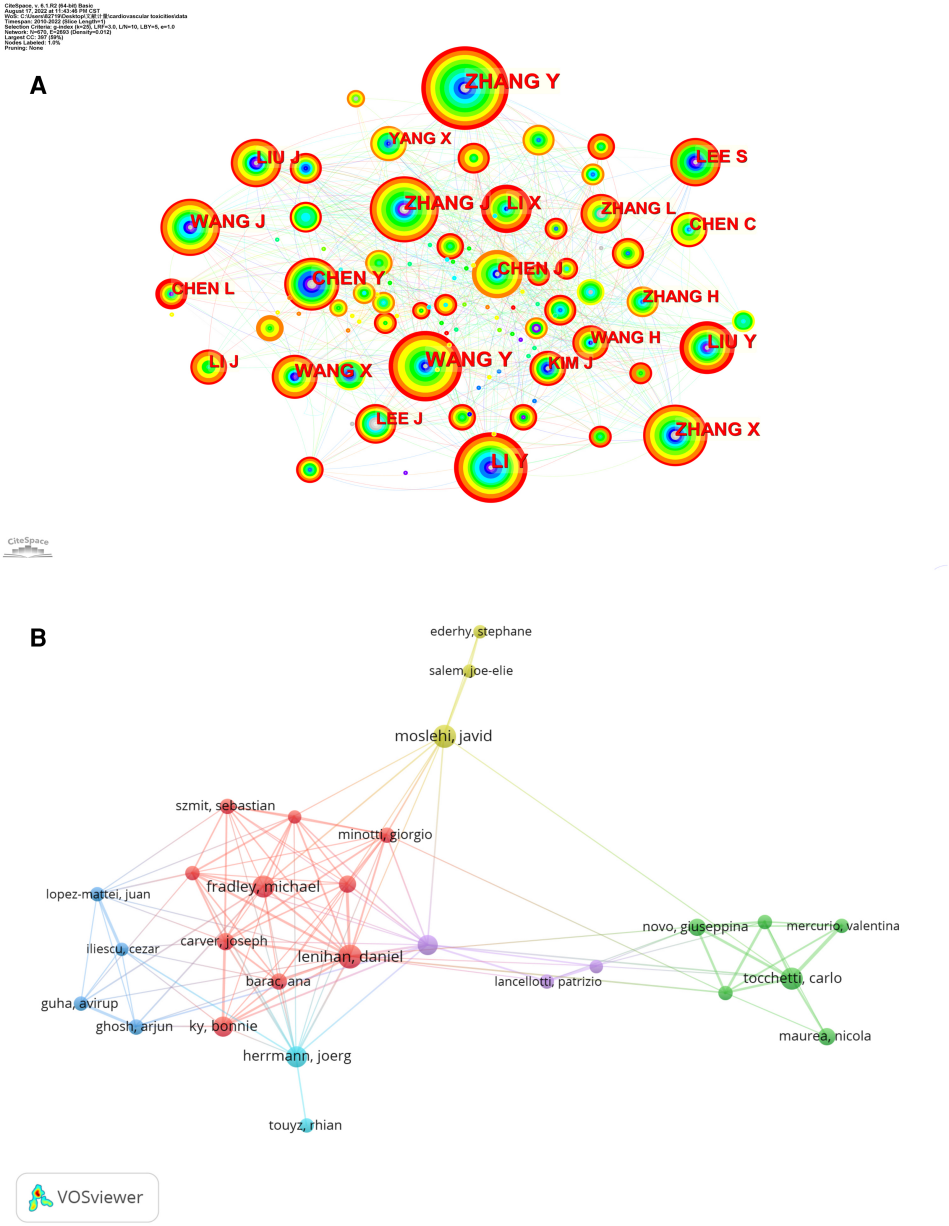
(**A**) citeSpace visualization map of authors involved in cardiotoxicity. (**B**) Network map of co-cited authors analysis based on VOS viewer.

The number of authors was 39,071, and there were 8,074 publications that they had co-authored. Table 3 shows that Zhang, Yun had authored the most publications (*n* = 53), followed by Wang, Yue (*n* = 42), Li Ying (*n* = 38), and Sun, Zhiwei (*n* = 35) and Zhang, Jing (*n* = 34). There was a lack of centrality among the top 10 authors. None were more significant than 0.10. Figure 4 shows a certain degree of collaboration between the authors. The circles represent different authors, and the lines between them represent collaboration between them; different colors represent different years, and thicker lines indicate closer cooperation.

**Table 3:** The top 10 authors and co-cited authors of cardiotoxicity research.

Rank	Auther	Count	Centrality	Co-cited-auther	Citation	TLS
1	Zhang, Yun	53	0.03	Moslehi, Javid	2230	709
2	Wang, Yue	42	0.03	Ky, Bonnie	1375	375
3	Li Ying	38	0.01	Vanholder, Raymond	1362	66
4	Sun, Zhiwei	35	0.01	Abdollahi, Mohammad	1251	264
5	Zhang, Jing	34	0.05	Lyon, Alexander R.	1112	574
6	Herrmann, J	32	0.01	Sun, Zhiwei	1073	881
7	Lyon, Alexander R.	32	0.01	Breccia, Massimo	992	52
8	Liu, Ying	31	0.02	Duan, Junchao	956	752
9	Duan, Junchao	29	0.00	Schepers, Eva	942	52
10	Li, Jing	28	0.00	Plana, Juan Carlos	937	271

TLS, Total linkstrength.

Co-cited authors are authors whose papers are cited by one or more other publications at the same time. There were more than 900 citations among the top 10 co-cited authors, as shown in Table 3. Moslehi, Javid (*n* = 2,230) and Ky, Bonnie (*n* = 1,375) were the most frequently co-cited authors, followed by Vanholder, Raymond (*n* = 1,362), Abdollahi, Mohammad (*n* = 1,251) and Lyon, Alexander R (*n* = 1,112). The minimum citation threshold of VOS viewer was set to 10 to produce a map with 60 nodes and 6 clusters (Figure 4B). Table 3, Figure 4B shows that co-cited authors with high TLS play an essential bridge role.

### Journals and co-cited journals

In order to find the journals with the most co-citations and published papers, we used the VOS viewer and Citespace software. There were 8,074 papers published in 2,392 academic journals, according to the results. The most published articles are Plos One (*n* = 93), followed by Cardiovascular Toxicology (*n* = 81), Frontiers in pharmacology (*n* = 76), Clinical Toxicology (*n* = 68), and Journal of Ethnopharmacology (*n* = 68), as shown in Table 4. There were nine journals in the top 10 that published more than 50 papers, seven of which were in the Q1 category of Journal Citation Reports (JCR). Furthermore, Chemosphere (IF = 8.94) has the highest impact factor (IF) of all these journals.

**Table 4:** Top 10 journals related to cardiotoxicity.

Rank	Journal	Count	IF (2021)	JCR (2020)
1	Plos One	93	3.752	Q2
2	Cardiovascular Toxicology	81	2.755	Q2
3	Frontiers in pharmacology	76	5.988	Q1
4	Clinical Toxicology	68	3.738	Q2
5	Journal of Ethnopharmacology	68	5.195	Q1
6	Toxicological Sciences	67	4.109	Q1
7	Chemosphere	51	8.943	Q1
8	Environmental Pollution	50	9.988	Q1
9	Science of The Total Environment	50	10.753	Q1
10	Scientific Reports	49	4.996	Q1

IF, Impact Factor; JCR, Journal Citation Reports.

**Figure 5:**
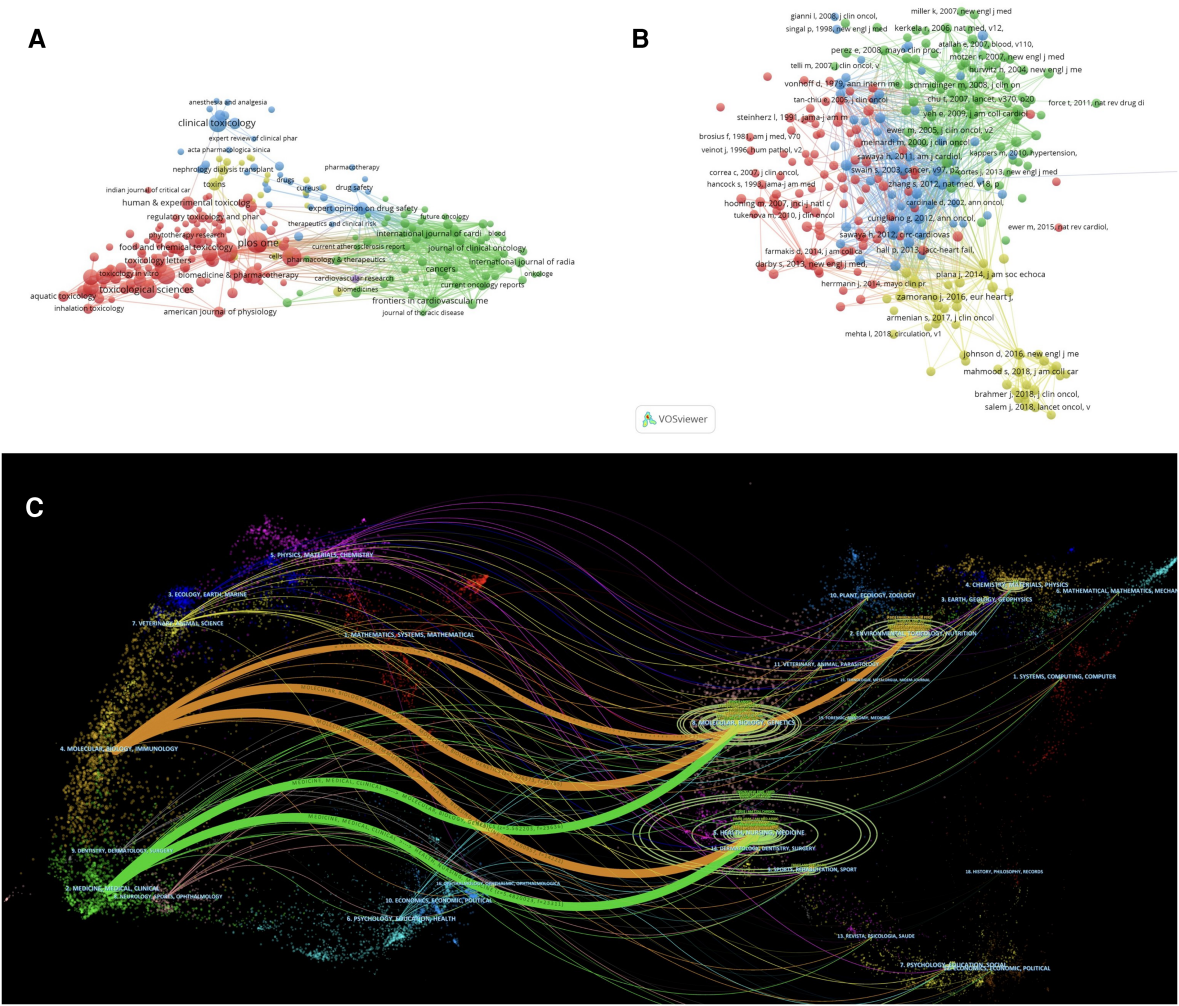
(**A)** network map of journals analysis based on VOS viewer. (**B**) Network map of co-cited journals analysis based on VOS viewer. (**C**) A dual-map overlay of the journals on cardiotoxicity research generated by using CiteSpace software. In the dual-map, the citing journals appear on the left side of the map, and the cited journals are on the right. The wider lines that begin from the citing journals and end at the cited journals represent the main citing pathways calculated from the so-called z-score of the citation links.

According to Table 5, the most frequently cited journals were the New England Journal of Medicine (*n* = 10,674), followed by Journal Of Clinical Oncology (*n* = 10,063), Journal of Circulation (*n* = 9,263), Journal of The American College of Cardiology (*n* = 5,501), and Lancet (*n* = 5,404). There were six journals with more than 5,000 citations among the top 10 co-cited journals, and New England Journal of Medicine had the most citations overall. The journals with the highest IF were the New England Journal of Medicine (IF = 176.079) among them, followed by Lancet (IF = 202.731), Journal of the American Medical Asociation (IF = 157.335), Journal of Clinical Oncology (IF = 50.717), and Circulation (IF = 39.918). Figure 5A shows the citation patterns of five journals, divided into 4 clusters with 399 links. In Figure 5B, which contains 1,000 items and 7 clusters, the co-citation relationship among different journals is visualized.

**Table 5:** Top 10 co-cited journals related to cardiotoxicity.

Rank	Journal	Citation	IF (2021)	JCR (2021)
1	New England Journal of Medicine	10,674	176.079	Q1
2	Journal of Clinical Oncology	10,063	50.717	Q2
3	Circulation	9263	39.918	Q1
4	Journal of The American College of Cardiology	5501	27.203	Q1
5	Lancet	5404	202.731	Q1
6	Plos One	5181	3.752	Q2
7	Journal of Biological Chemistry	4887	5.486	Q2
8	Proceedings of The Nnational Academy of Sciences of the United States of america	4242	12.779	Q1
9	Environmental Health Perspectives	4185	11.035	Q1
10	Jama journal of The American Medical Asociation	3569	157.335	Q1

IF, Impact Factor; JCR, Journal Citation Reports.

The dual-map overlay of journals shows how journals are distributed and how they relate to cited journals (the color path shows the relationship between journals and cited journals). Linked journal citations are displayed on the left, followed by cited journals on the right, with colored paths indicating relationships among the journals. Five main reference paths are shown in Figure 5C. It showed that studies published in “Environment, Toxicology, nutrition”, “Molecular, Biology, Genetics” and “Health, Nursing, Medicine” journals were frequently referenced in articles published in “Molecular, Biology, Immunology” journals and “Medicine, medical, Clinical” journals.

### Keyword co-occurrence, clusters, and burst

**Figure 6:**
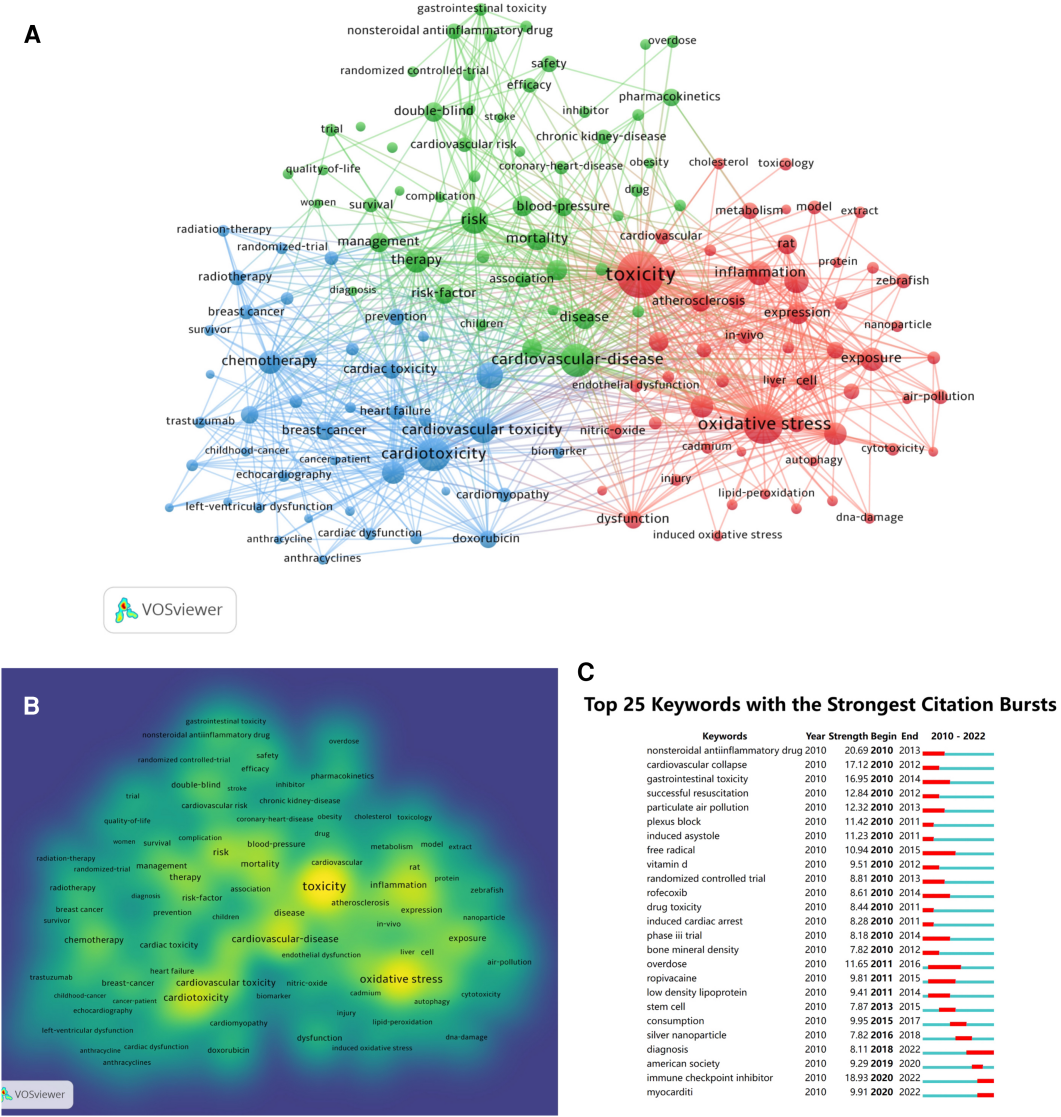
(**A)** keyword co-occurrence analysis on cardiotoxicity research using the VOS viewer. (**B**) Density map of keywords generated by the VOS viewer. The part has the hottest color in the density map, indicating that they were the most prevalent elements. (**C**) Top 25 keywords with the strongest citation bursts.

In order to identify the most popular hot spots in a specific field of study, keywords are often used to indicate their frequency of co-occurrence, and the approach is critical for tracking scientific progress. There were 29,533 keywords extracted from 8,074 papers in total. Co-occurrence frequency for the top 10 keywords from WoSCC is shown in Table 6. Using 150 keywords with more than 20 co-occurrences, we created a density map, with which hot topics can be intuitively visualized in the field as a whole. According to Figure 6B, “toxicity”, “oxidative stress”, “cardiovascular disease” and “cardiotoxicity” had the hottest color in the density map, indicating that they were the most prevalent elements.

**Table 6:** The top 10 keywords in co-occurrence frequency of cardiotoxicity.

Rank	Keyword	Occurrence	TLS
1	Toxicity	1527	4006
2	Oxidative stress	1125	3608
3	Cardiovascular-disease	765	2188
4	Cardiotoxicity	755	3133
5	Risk	544	1878
6	Cardiovascular toxicities	499	1850
7	Cancer	481	1698
8	Therpy	432	1515
9	Inflammation	426	1528
10	Chemotherapy	410	967

TLS, Total linkstrength.

A cluster analysis was performed on the selected keywords. The index words derived from the keywords were used to mark all clusters. In Figure 6A, 150 high-frequency keywords are grouped into three clusters, which are representative of the three dominant lines of research in the field. With 56 keywords marked in red circles, cluster 1 is the largest cluster, focusing on mechanisms of cardiotoxicity, including “toxicity”, “oxidative stress” and “inflammation”. There are 52 keywords in cluster 2, marked with blue circles, which focus on cardiotoxicity diseases. Among keywords were “heart-failure”, “cancer” and “breast-cancer”. A green circle was drawn around cluster 3, which included 42 keywords relating to risk factors, including “overdose”, “blood pressure”, and “therapy”.

Additionally, we detected keywords with the most citation bursts, which can indicate trends or frontiers in research in recent years. Among them, Figure 10 shows the top 25 keywords. As shown in Figure 6C, the following is a list of keywords that experienced a citation burst after 2017: stem cell (7.87), consumption (9.95), sliver nanoparticle (7.82), diagnosis (8.11), immune checkpoint inhibitor (18.93) and myocarditi (9.91), which were the frontiers of cardiotoxicity.

### References and co-cited references

**Figure 7:**
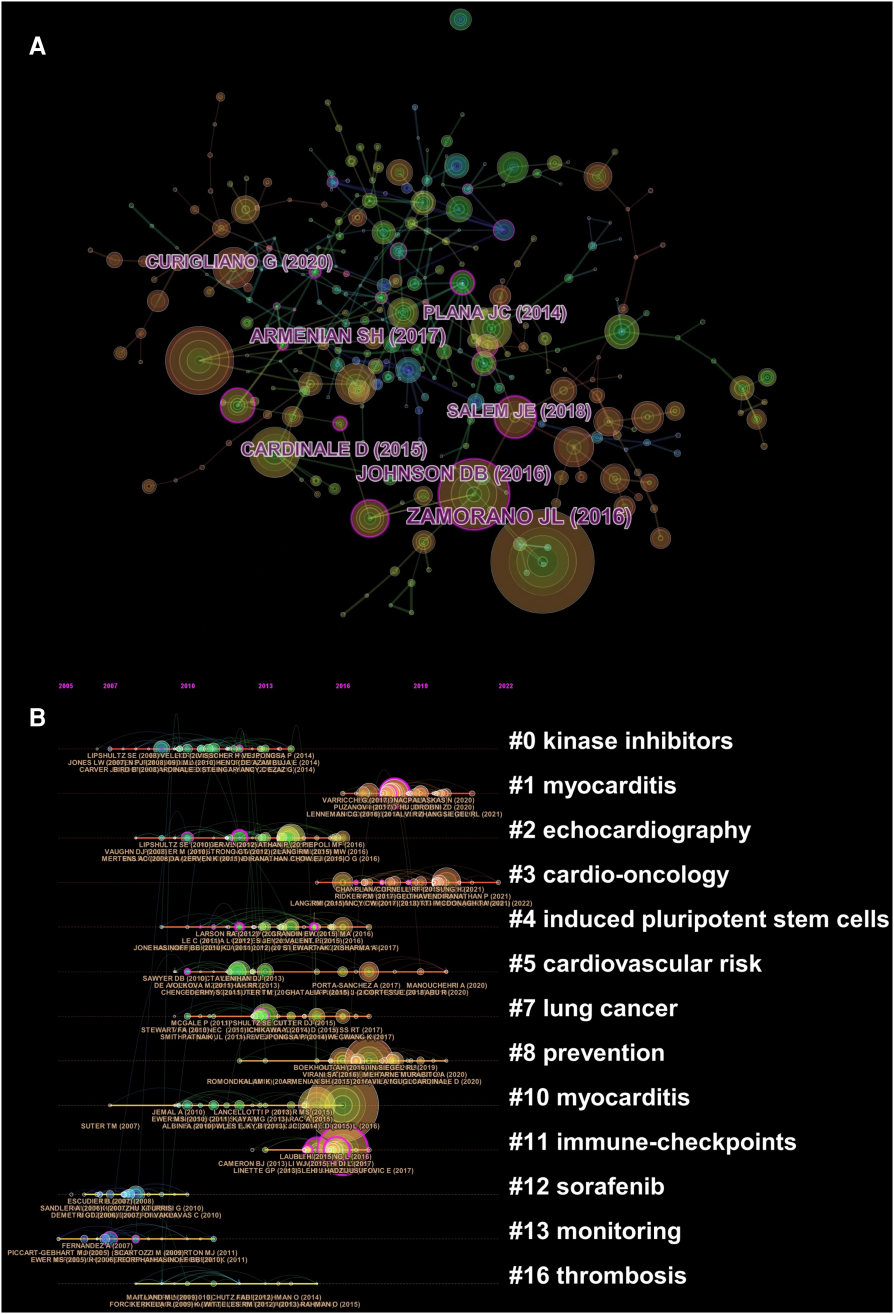
(**A)** the citation map of reference in cardiotoxicity. (**B**) Timeline view of co-cited references of cardiotoxicity by using CiteSpace software. Thirteen cluster tags representing hot themes were selected and ranked on the right of Figure based on the loglikelihood ratio (LLR) algorithm. Along the dashed line linked to each tag, circles with larger radius represented higher citation frequencies and warmer colored lines represented later publication dates.

Figure 7A shows the top 10 most cited references (27–36) over the course of the study, which we analyzed further to understand the development trends. As shown in Table 7, there were co-cited at least 70 times among the top 10 co-cited references. The 2016 ESC position paper on cancer treatments and cardiovascular toxicity, which received 191 citations, was among the most deeply cited. In addition, there are nine research articles and one review article among the top 10 articles. Furthermore, it reflects the fact that professional association guidelines are commonly quoted papers within a discipline because they are closely related to medical practice. Commonly, citation rates are correlated with the time of publication, so current citation counts may not match their current value.

**Table 7:** The top 10 cardiotoxicity references with the most citations.

Auther	Title	Journal	Citation	Centrality
Zamorano, JL	2016 ESC Position Paper on cancer treatments and cardiovascular toxicity developed under the auspices of the ESC Committee for Practice Guidelines	European Heart Journal	191	0
Johnson, DB	Fulminant Myocarditis with Combination Immune Checkpoint Blockade	New England Journal of Medicine	131	0.22
Armenian, SH	Prevention and Monitoring of Cardiac Dysfunction in Survivors of Adult Cancers: American Society of Clinical Oncology Clinical Practice Guideline	Journal of Clinical Oncology	128	0.02
Cardinale D	Early Detection of Anthracycline Cardiotoxicity and Improvement With Heart Failure Therapy	Circulation	96	0.02
Plana JC	Expert Consensus for Multimodality Imaging Evaluation of Adult Patients during and after Cancer Therapy: A Report from the American Society of Echocardiography and the European Association of Cardiovascular Imaging	Journal of the American Society of Echocardiography	79	0.03
Curiglianog	Management of cardiac disease in cancer patients throughout oncological treatment: ESMO consensus recommendations	Annals of Oncology	76	0.01
Salem JE	Cardiovascular toxicities associated with immune checkpoint inhibitors: an observational, retrospective, pharmacovigilance study	Lancet Oncology	76	0.23
Gulati G	Prevention of cardiac dysfunction during adjuvant breast cancer therapy (PRADA): a 2 × 2 factorial, randomized, placebo-controlled, double-blind clinical trial of candesartan and metoprolol	European Heart Journal	75	0.08
Mahmood SS	Myocarditis in Patients Treated With Immune Checkpoint Inhibitors	Journal of the American College of Cardiology	75	0.05
Moslehi JJ	Cardiovascular Toxic Effects of Targeted Cancer Therapies	New England Journal of Medicine	70	0.02

In the references timeline view, you can see how research hotspots evolved over time. Cluster labels were determined by identifying the terms that appeared most frequently in each cluster. As shown in Figure 7B, The cluster 0 (kinase inhibitor) and cluster 12 (sorafenib) appeared first, suggesting that clinical medicine was the primary focus early on. The cluster 7 (lung cancer), cluster 10 (myocarditis) and cluster 11 (immune-checkpoints) occurred in 2010–2019. Cluster 3 (cardio-oncology) appeared in 2014–2022. This indicates that cardio-oncology is a popular topic of current research, suggesting that greater attention is being paid to this area.

## Discussion

### General information

This bibliometric analysis of the 8,074 cardiotoxicity-related documents in the WOSCC database for the past 12 years revealed current status, research frontiers and trends in the cardiotoxicity field. Research on cardiotoxicity was in a state of rapid development. Over the past 12 years, the number of publications on cardiotoxicity steadily increased worldwide. There have already been 496 papers published in 2022 so far, which has almost reached half of last year's total, and the outputs were expected to hit a new all-time high. Another obvious advantage of bibliometric analysis provides the ability to visualize global trends from countries/regions, institutions, authors, and journals over time (37).

Table 1 and Figure 2 showed that the United States was the world leader country in cardiotoxicity research, which had accumulated the highest number of critical citations. Noticeably, despite its late start, China is now one of the top productive countries in the world. Besides, Figure 2 showed that active collaborations among different countries, indicating cardiotoxicity had gained worldwide interest, and the United States was the key collaborating center. Italy and England showed the considerable increases in output in recent years, collaborating most closely with the United States.

In our analysis, the top 10 productive institutions were from four different countries, and 7 (70%) were in the United States, with the top 2 institutions respectively from the United States and Canada. In 2000, the University of Texas MD Anderson Center, a famous pioneer, established the first cardio-oncology unit (38), and it is the most prolific institution publishing academic research related to cardiotoxicity. This means that cardiotoxicity is more likely to be noticed by oncologists, mainly due to the treatment of patients with cancer. Nevertheless, as an interdisciplinary field, cardiotoxicity requires more multidisciplinary collaboration and treatment. Moreover, we found no oncology organization among actively collaborating institutions, including the Vanderbilt University, Harvard Medical School, and Mayo Clinic. To prevent incidence of cardiotoxicity, countries and institutions may need to strengthen their cooperation in cardiotoxicity research to further advance the development of cardio-oncology.

By highlighting the contributions of the most productive researchers in a given field, researchers in this field could be inspired by understanding these pioneer researcher's work (39). The top 5 productive authors were from China, while top 5 co-cited authors were all from other countries. Furthermore, we found three researchers, namely Sun, Zhiwei, Lyon, Alexander R., and Duan, Junchao were among both the top 10 most productive authors and the top 10 most co-cited authors, implying that these three authors had an outstanding contribution to cardiotoxicity field.

### The development of cardio-oncology

In this analysis, we found that in 2017, the most co-cited study was published (*n* = 191) by European Heart Journal co-authored by Jose Luis Zamorano and 17 other outstanding researchers in cardiotoxicity research (27). This practice guideline focuses on cardio-oncology, calling for the awareness and recognition of cardiotoxic side effects during cancer treatment. Since then, countries around the world have gradually begun to recognize and paid attention to the cardiotoxicity related to tumor therapy. Johnson et al. published the second most co-cited paper in the New England Journal of Medicine in 2017 (28). The study reported two cases of melanoma patients who developed fatal myocarditis following combination immunotherapy. Characterizing these severe cardiotoxicity is a major priority, despite the durable antitumor effect produced in cancer patients by combination immune checkpoint inhibition. The mechanism behind its cardiotoxicity has become a research hotspot. Following the European Society of Cardiology Committee, in 2017, the American Society of Cardio-oncology released a similar clinical practice guideline for prevention and monitoring of cardiotoxicity in survivors of adult-onset cancers, which is the third co-cited publication (29). Subsequently, cardio-oncology was well recognized as a new area in the US and most European countries. Daniela Cardinale et al. published the fourth co-cited article in Circulation in 2015 (30). This study prospectively evaluated the incidence, timing, clinical relevance, and response to heart failure therapy of cardiotoxicity following anthracycline-containing therapy and demonstrated that early detection and prompt treatment of cardiotoxicity are critical for substantial recovery of cardiac function. Juan Carlos Plana et al. published the fifth co-cited articles in 2014 (31). This review introduced Definition of Cancer Therapeutics–Related Cardiac Dysfunction (CTRCD), and summarizes the recommended cardio-oncology-echocardiogram protocol.

Overall, the top 5 co-cited references focus on field of cardio-oncology. With the tremendous development of cardio-oncology, more attentions are paid in detection, monitoring, prevention and therapy of cardiovascular diseases(CVDs) occurring in the context of cancer treatment (40). Meanwhile, immune-based therapies have revolutionized cancer treatments, however, the serious and fatal cardiotoxic effects, representing new challenges in cardio-oncology (41).

The top 5 papers were published in 2014–2017 (the top 3 in 2017). This is a critical period for the start and development of cardio-oncology, which suggests that cardiovascular toxicities have become research hotspot and are attracting increasing attention. This year, the International Cardio-Oncology Society published consensus statement seeking to provide a reliance for defining cardiovascular toxicities of cancer therapy including cardiomyopathy/heart failure, myocarditis, vascular toxicity, hypertension, as well as arrhythmias and QTc prolongation (42). It means that tumor cardiotoxicity has become an international public topic, and we believe it will be a high co-cited study. These results of highly co-cited references may be explained by the fact that advances in treatment have led to improved survival for cancer patients.

### Research trend and frontiers

#### Hotspot evolution

In bibliometrics, keywords co-occurrence frequency of cardiotoxicity (Figure 5 and Table 6) reveals the focus of this research field, and Figure 7 shows the evolution of new hotspots of the timezone view (43). Growing terms in the early phase (2010–2013) included drug toxicity, such as nonsteroidal anti-inflammatory drug, particulate air pollution and severe degree of cardiotoxicity, including induced cardiac arrest and cardiovascular collapse etc. While during the stable-growth phase (2014–2018), new terms contained more mechanisms explored and focus on different diseases, including gastrointestinal toxicity, overdose, ropivacaine, silver nanoparticle, low density lipoprotein, etc. Notably, doxorubicin consumption is becoming ever more prevalent for long-term cardiovascular complications (44). In the rapid development stage (2019-now), as oncology treatment has changed dramatically, emerging topics, like immune checkpoint inhibitor, stem cell, and myocarditis imply that immune-based therapies have revolutionized cancer treatment (45). Meanwhile, cardiotoxicity is a dynamic variable, which can be influenced by several variables, including optimization of pre-existing CVDs, dosage and frequency of medication, and duration of oncology treatment, implementation of primary prevention treatments, the overall cumulative treatment received, the time since treatment, and the interaction with other CVDs (46). The potential methods both biomarkers and imaging-based approaches to diagnose and predict cardiotoxicity earlier are evaluated (47).

### Emerging topics of cardiotoxicity

The cluster of keywords could fully demonstrate frontiers of academic research, and every cluster reflected a different research topic (48). The cardiotoxicity field is divided into three main clusters (Figure 6) by cluster analysis, including the mechanisms, the related disease, and risk factors of cardiovascular toxicities, representing three Emerging aspects of cardiotoxicity research.

#### The molecular mechanism of cardiovascular toxicities

As shown by the keyword co-occurrence analysis, one of the hotspots in cardiotoxicity research is potential mechanisms and correspondingly potential targets for prevention and treatment of cardiotoxicity. Oxidative stress-triggered cellular events are central mechanisms of doxorubicin (DOX) which has long been known as the mainstay of anticancer drug-induced cardiotoxicity (49). Besides, oxidative stress also plays a key role in radiation-induced cardiovascular damage (50). Therefore, antioxidant strategies have become an important therapeutic target (51). Other mechanisms, such as systemic inflammation, endothelial injury and neutrophil recruitment, have been identified as trigger of the progression of DOX-induced cardiomyopathy and could be promising target for cardiotoxicity prevention and treatmen (52). In addition, the search for more effective therapies with minimal off-target side effects is of urgent need. Photodynamic therapy (PDT), a non-invasive cancer therapeutic modality with clinical appeal, has received increasing attention for their advantages of high selectivity and low cytotoxicity (53).

#### The related disease of cardiovascular toxicities

As shown in cluster 2, the related disease of investigations have focused on heart failure (HF) and cancer. Cancer-induced cardiomyopathy results in cardiac dysfunction, similar to congestive heart diseases, which is accompanied by cardiac atrophy, metabolic remodelling, fibrosis and changes in the ultrastructure of the heart (54). Cancer survivors may develop HF as a result of chemotherapy, radiotherapy and immunotherapy, often in combination (55). Therefore, the future development trend is to establish multidisciplinary medical teams, optimize the treatment strategies of HF patients with a history of cancer, and promote the clinical implementation of precision medicine strategies.

#### The risk factors of cardiovascular toxicities

The risk factors in cluster 3 include “blood pressure” and “dose”, which could reflect the current research focus. Management of these two risk factors remains the research hotspot in the current and future. By improving monitoring and management of hypertension before, during, and after cancer treatment, cardiovascular risks can be minimized (56). This is vital to optimize cardiovascular health in patients with cancer survivors, and to ensure that advances in terms of cancer survivorship do not come at the expense of increased cardiovascular toxicities.

### Strategies for surveillance and prevention

Advances in antineoplastic agents research have led to huge improvements in malignant tumors survival rates. Meanwhile, cancer treatments, including cytotoxic chemotherapy, molecular target therapy, and mediastinal site radiotherapy, are thought to be linked with cardiomyocyte ischemia, damage, conduction and rhythm disturbances, ventricular dysfunction, cardiac failure, and several other cardiovascular complications (57). The mechanisms of toxicities caused by antitumor agents on the cardiovascular system are diverse and not fully understood, including direct effects on the heart or vascular system, release of cardiac regulators, oxidative stress and changes in blood coagulation status. Therefore, in this era of treatment, new models for multidisciplinary support team including cardiologists and oncologists are required and attention should be paid to the regular monitoring of patients' heart function during tumor therapy to help mitigate associated cardiovascular complications (32).

However, the available options for cardiac protection are minimal, and current medications to combat chemotherapy-induced side effects generally fail to address the underlying cause and create distress for patients undergoing therapy. In the future we need to discover the different mechanisms of action of chemotherapeutic agents so that the toxicities can be circumvented along with improved clinical experience for patients.

## Conclusion

This bibliometric analysis provides a thorough analysis of the cardiotoxicity from 2010 to 2022. Our results show that the United States is the world leader country in cardiotoxicity research, while China is now developing rapidly. This analysis reveals a strong relationship between cardiology and oncology in the development of cancer treatment. The interdisciplinary property of cardio-oncology has developed rapidly in the past decade, and as shown in this bibliometrics study, cardiotoxicity or cardio-oncology has not been thoroughly studied yet, which provides both opportunities and challenges.
